# Thymidylate synthase expression as a predictive biomarker of pemetrexed sensitivity in advanced non-small cell lung cancer

**DOI:** 10.1186/s12890-015-0132-x

**Published:** 2015-10-26

**Authors:** Cristina Chamizo, Sandra Zazo, Manuel Dómine, Ion Cristóbal, Jesús García-Foncillas, Federico Rojo, Juan Madoz-Gúrpide

**Affiliations:** Cancer Biomarkers Research Group, Fundacion Jimenez Diaz University Hospital Health Research Institute, UAM, Madrid, Spain; Translational Oncology Division, Oncohealth Institute, Fundacion Jimenez Diaz University Hospital, Madrid, Spain

**Keywords:** Thymidylate synthase, NSCLC, Pemetrexed

## Abstract

**Background:**

Although it has been suggested that a high level of thymidylate synthase (*TYMS*) gene expression in malignant tumors is related to reduced sensitivity to the antifolate drug pemetrexed, no direct evidence for such an association has been demonstrated in routine clinical samples from patients treated with the drug. The purpose of this study was to quantitatively assess the impact of *TYMS* gene expression in tumor cells as a predictor of the efficacy of pemetrexed therapy in patients with advanced non-small cell lung cancer (NSCLC) treated at our institution.

**Methods:**

Sixty-two NSCLC patients were included in this study: 16 patients received platins-pemetrexed as first-line NSCLC, and 46 pemetrexed in monotherapy as second- or subsequent-line treatment. Total mRNA was isolated and the expression of *TYMS* was analyzed by RT-qPCR. *TYMS* levels were calibrated against expression in normal lung tissue.

**Results:**

*TYMS* overexpression was detected in 61 % of patients and low expression in 39 %. The response rate for patients with low *TYMS* expression was 0.29 compared with 0.03 in patients with overexpression (*P =* 0.025). A significant benefit was observed in patients with low expression both in time to progression (average TTP = 56 vs. 23 months, *P =* 0.001) and in overall survival (average OS = 60 vs. 25 months, *P =* 0.002).

**Conclusions:**

*TYMS* overexpression in tumor cells correlated with a reduced response to pemetrexed-containing chemotherapy and might be used as a predictive biomarker in advanced NSCLC patients.

## Background

Pemetrexed, an analogue of folic acid (folate), is a folate antimetabolite agent that shows antitumor activity, inhibiting 3 enzymes involved in *de novo* purine and pyrimidine synthesis: thymidylate synthase (TYMS), dihydrofolate reductase, and glycinamide ribonucleotide formyltransferase [1]. Consequently, pemetrexed inhibits DNA and RNA biosynthesis. This agent inhibits the cellular growth of a variety of tumor types and has been approved for non-small cell lung cancer (NSCLC) at locally advanced and metastatic stages [2] for first- and second-line therapy.

As pemetrexed inhibits TYMS more effectively than the rest of the folate-dependent enzymes, most studies have focused on the effects of pemetrexed on TYMS. In vitro studies have demonstrated that high baseline expression levels conferred resistance to pemetrexed [3–5]. Similarly, some clinical studies have associated elevated TYMS expression levels with poorer chemotherapeutic response to pemetrexed, including breast cancer [6], colorectal cancer [7], head and neck cancer [8], and malignant pleural mesothelioma [9]. In a large phase III study in advanced-stage NSCLC patients, survival differences were reported if favor of a cisplatin/pemetrexed regimen compared to cisplatin/gemcitabine according to histology [10]. This was explained by a previous paper showing that the baseline expression of the thymidylate synthase gene and protein were significantly higher in squamous cell carcinoma compared with adenocarcinoma (*P* < 0.0001) [11]. According to some published reports, elevated expression of TYMS may be predictive of sensitivity to pemetrexed-based chemotherapy. However, in some cancer types, such as advanced NSCLC, this point is controversial. For this reason, we evaluated the relationship between *TYMS* gene expression and clinical outcome in a cohort of 62 patients with advanced NSCLC treated with a pemetrexed-based regimen at our institution. A quantitative real-time PCR (qPCR) assay was devised to determine the *TYMS* gene expression level. qPCR is suitable for use with mRNA from archived formalin-fixed, paraffin-embedded (FFPE) samples, as it amplifies <100-bp amplicons. Additionally, it is faster and more precise than immunohistochemistry (IHC). And it has been reported a correlation between *TYMS* mRNA levels and protein abundance [9]. However, both techniques must be standardized before consistent comparisons can be made when interpreting retrospective/prospective studies.

In conclusion, *TYMS* overexpression correlated with response to pemetrexed and death, and a significant benefit was observed in patients with low *TYMS* expression, suggesting that this enzyme might be used as a predictive biomarker in advanced NSCLC patients.

## Methods

### Patient samples

A single-institution retrospective analysis was carried out including samples archived in the Fundacion Jimenez Diaz Biobank (Madrid) from 62 consecutive patients who had received clinical follow-up from. The study included 62 patients with stage IV NSCLC (49 adenocarcinomas, 7 NSCLC nos and 6 squamous-cell carcinomas). Sixteen patients received platins-pemetrexed as first-line treatment for NSCLC and 46 received pemetrexed as monotherapy in second and subsequent lines. Tissue microarrays were constructed with 3 1.0-mm cores obtained from FFPE tumor biopsies before treatment. Immunostaining was performed to discriminate between histological subtypes. The study was approved by the hospital ethics committee and was conducted in accordance with institutional guidelines.

### Ethics statement

The study was approved by the ethics committee of the Fundacion Jimenez Diaz hospital (CEIC-FJD) in accordance with the Spanish Royal Legislative Decree RD 223/2004.

### Consent statement

Written informed consent for participation in the study was obtained from all participants.

### Gene expression analysis by qPCR

The level of *TYMS* gene expression was determined by a quantitative RT-real time PCR assay on 5 × 10-μm sections of the FFPE biopsies using *ATP5E* as a housekeeping gene. Total RNA was isolated using the RNeasy FFPE kit (Qiagen). Primers were designed according to the mRNA sequences NM_00101071 for TYMS (and NM_006886.2, and NM_001001977.1 for ATP5E. qPCRs were performed using the LightCycler480 II system (Roche Applied Science, Switzerland) for 45 cycles with the following sets of primers: TYMS, 5′-CCTCTGCTGACAACCAAACG (exon 1) and 5′-GAAGACAGCTCTTTAGCATTTG (exon 2); ATP5E, 5′-CCGGCGTCTTGGCGATTC (exon 1) and 5′-GATCTGGGAGTATCGGATG (exon 2).

**Fig. 1:**
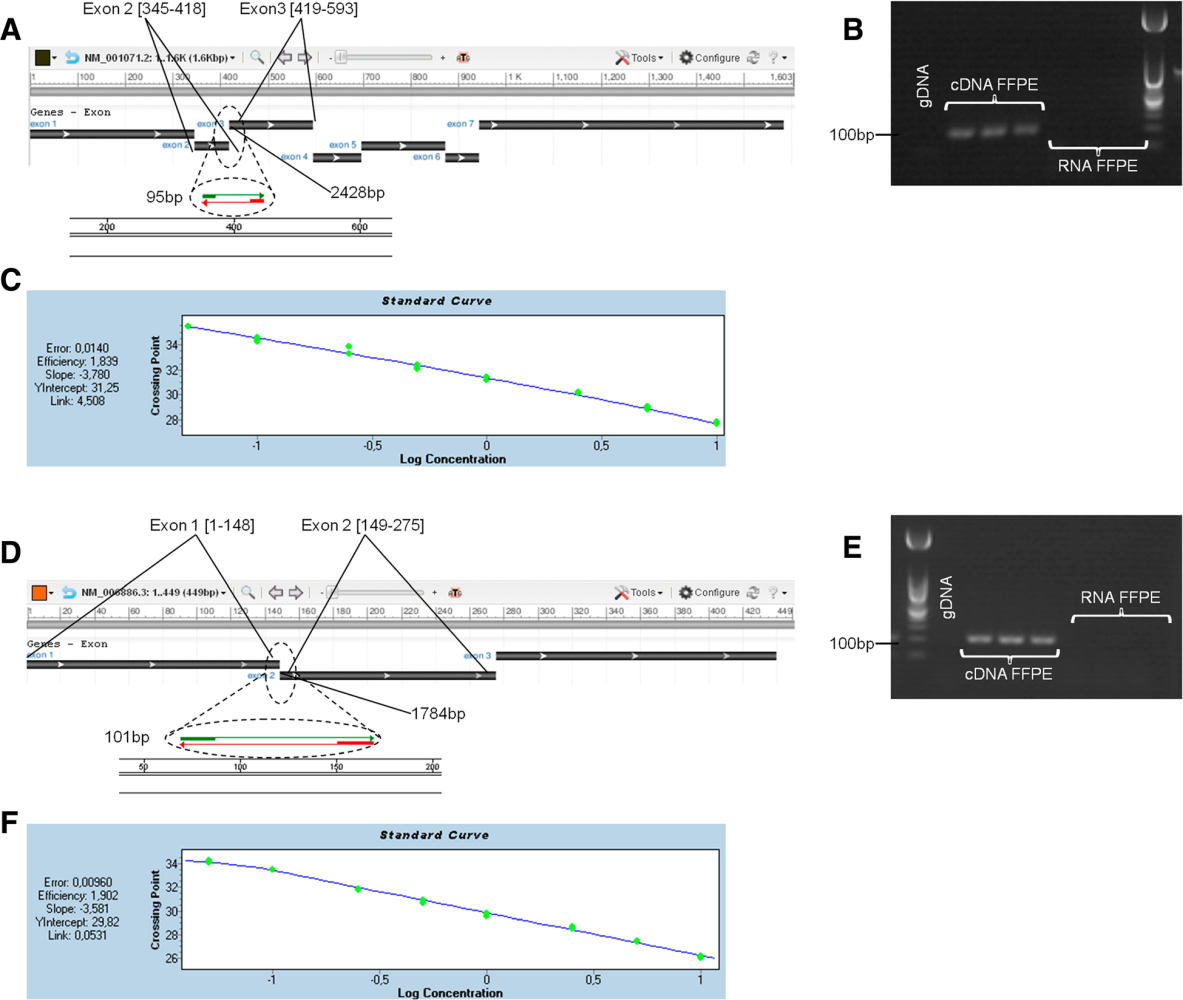
Design and optimization of the qPCR assay. **a** Primer region selection for *TYMS* target gene used for expression analysis. NCBI Reference Sequence was NM_001071.2 (Homo sapiens thymidilate synthetase, mRNA). **b** Specificity for TYMS mRNA sequence was demonstrated in a 2 % agarose gel electrophoresis loaded with 10 μl PCR products from 3 random FFPE samples. TYMS products are expected to be 95 bp long. DNA Molecular Weight Marker XIII 50 bp ladder (Roche). **c** Primer efficiency for the TYMS qPCR assay. The efficiency of the primer pair was assessed by plotting the cycle threshold value (Cp) at each concentration against the logarithm of the fold dilution of the sample. The slope of a linear-regression trend line is indicative of primer efficiency. **d** Primer region selection for *ATP5E* reference gene. NCBI Reference Sequence was NM_006886.3 (Homo sapiens ATP synthase, H+ transporting, mitochondrial F1 complex, epsilon subunit, mRNA). **e** Specificity for ATP5E mRNA sequence was demonstrated in a 2 % agarose gel electrophoresis loaded with 10 μl PCR products from 3 random FFPE samples. ATP5E products are expected to be 101 bp long. **f** Primer efficiency for the ATP5E qPCR assay

Relative *TYMS* expression ratios were calculated using the Pfaffl method [12], using the *ATP5E* levels as the reference sample. *TYMS* expression levels were normalized to the calibrator levels (normal lung tissue) (Fig. 1). The efficiencies of every primer pair were estimated by a standard curve.

### Statistical analysis

The primary end points were objective response rate, time to progression (TTP), and overall survival (OS). TTP was defined as the time from treatment to the start of progression, censored at last contact. OS was defined as the time elapsed from the date of initial diagnosis to the date of death from any cause or the date of last follow-up. Receiver operating characteristic (ROC) analysis was used to determine the optimal cutoff value based on progression endpoint for *TYMS*. Survivals were analyzed by the Kaplan-Meier method (median follow-up, 75 months) and curves were compared by the log-rank test. Multivariate analysis including continuous quantitative and qualitative clinical-pathologic parameters was done using the Cox proportional hazards model. All statistical tests were conducted at the two-sided 0.05 level of significance. This work was performed in accordance with the Reporting Recommendations for Tumor Marker Prognostic Studies (REMARK) guideline. Statistical analysis was carried out using the IBM SPSS, version 21.0.

## Results

### *TYMS* gene expression in advanced tumors

In order to assess the suitability of *TYMS* gene-expression measurement as a predictive marker of pemetrexed sensitivity in advanced NSCLC patients, we first quantitatively evaluated the *TYMS* gene-expression levels in samples from the 62 pemetrexed-treated patients in our series. Patient data were normalized against healthy lung-tissue values. By performing a ROC curve analysis of *TYMS* expression against disease progression, we established a cutoff value of 2.55. Interestingly, 38 patients (61 %) were labeled as *TYMS*-overexpressing and 24 cases (39 %) showed low expression.

### *TYMS* overexpression is associated with disease progression

**Fig. 2:**
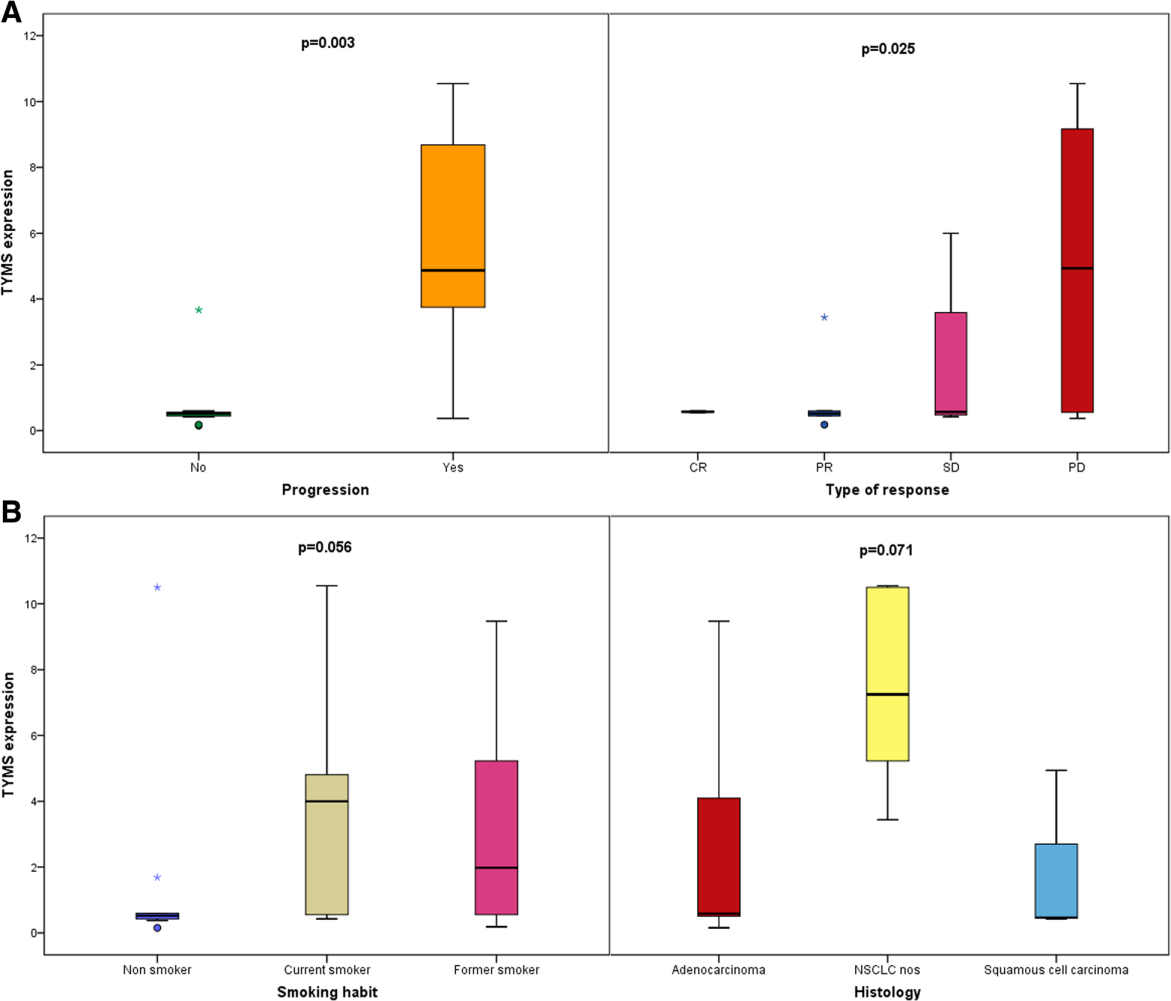
**a** Significant clinical-pathological correlations for *TYMS* expression in NSCLC patients. With respect to the type of response, tumors were categorized as either responding (CR, complete response or PR, partial response) or non-responding (SD, stable disease or PD, progressive disease). **b** Close-to-significance correlations

The clinical characteristics of the patients are shown in Table 1. *TYMS* gene-overexpression analysis was performed in 62 cases for which complete clinical records were available. This analysis showed significant correlation with progression (*P =* 0.003) and response to pemetrexed (*P =* 0.025) (Fig. 2a). We categorized the tumors as either responding or non-responding. The non-responding group showed significantly higher levels of *TYMS* expression as compared to the responding group. *TYMS* overexpression analysis also showed a tendency toward correlation between overexpression smoking (*P =* 0.056) and histology (*P =* 0.071) (Fig. 2b), but was not associated with gender, performance status, or line of treatment.

**Table 1:** Clinical-pathological correlations for *TYMS* gene expression in NSCLC patients treated with pemetrexed

		Total	*TYMS* high expression		*TYMS* low expression		
		n	n	%	n	%	*P*-value
Age (mean (range))		57 (32–79)	54 (32–79)		66 (60–78)		1
Gender	Male	40	26	65.0	14	35.0	0.419
	Female	22	12	54.5	10	45.5	
Smoking habit	Current smoker	24	18	75.0	6	25.0	0.056
	Former smoker	22	14	63.6	8	36.4	
	Never smoker	16	6	37.5	10	62.5	
Histology	Adenocarcinoma	49	27	55.1	22	44.9	0.071
	NSCLC nos	7	7	100	0	0	
	Squamous cell carcinoma	6	4	66.7	2	33.3	
ECOG performance status	ECOG0	27	19	70.4	8	29.6	0.392
	ECOG1	32	17	53.1	15	46.9	
	ECOG2	3	2	66.7	1	33.3	
Line of treatment	1st line	16	9	56.3	7	43.8	0.661
	2nd line	14	10	71.4	4	28.6	
	3rd, further lines	32	19	59.4	13	40.6	
Response	No	45	30	66.7	15	33.3	0.025
	Yes	7	1	14.3	6	85.7	
	Not evaluatable	10	7	70.0	3	30.0	
Progression	No	24	4	16.7	20	83.3	0.003
	Yes	38	34	89.5	4	10.5

### Low *TYMS* expression levels predict delayed progression in advanced cancers

**Fig. 3:**
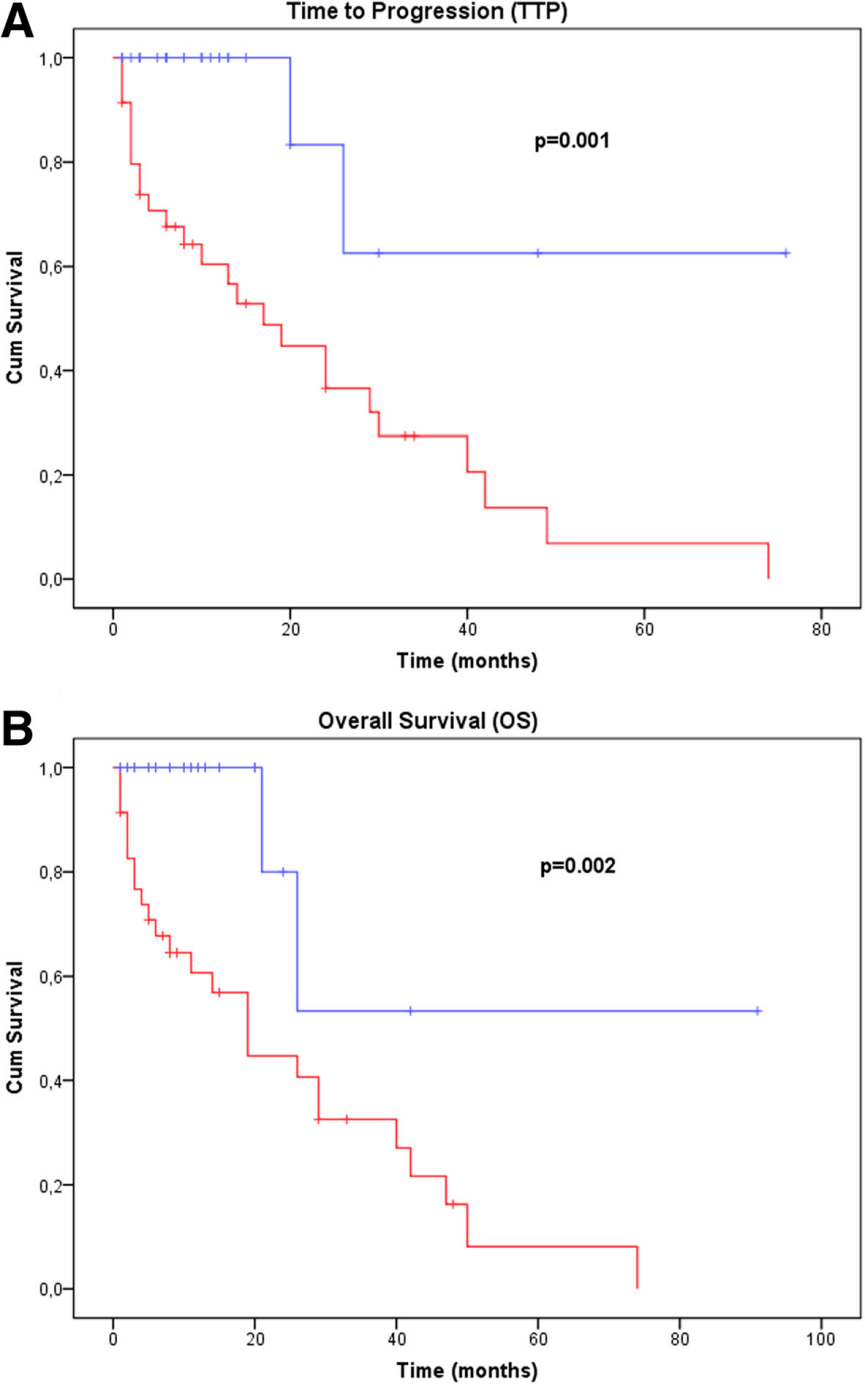
TTP (**a**) and OS (**b**) in NSCLC patients. The *blue line* denotes patients with low *TYMS* gene expression; the *red line* indicates patients with high *TYMS* expression

We found that patients with a low level of *TYMS* gene expression (cutoff < 2.55) had a significantly longer TTP than those with a high level (Fig. 3). NSCLC patients with low *TYMS* expression levels showed a significant benefit when treated with pemetrexed both in time to progression (average TTP = 56 vs. 23 months, *P =* 0.001) and in overall survival (average OS = 60 vs. 25 months, *P =* 0.002) (Fig. 3). These data thus suggest that *TYMS* expression level in advanced NSCLC tumors is inversely correlated with response to pemetrexed.

## Discussion

In the present study, we investigated the effects of *TYMS* gene overexpression on the sensitivity of advanced cancer cells to pemetrexed. Reliable predictive markers of beneficial therapy can aid in determining the most appropriate therapy for patients and minimize the negative effects of certain treatment regimens in non-responsive patients.

*TYMS* gene expression was determined by qPCR gene expression analysis in a series of NSCLC patients. Our results confirm the overexpression of *TYMS* in this population and suggest that assessment of *TYMS* gene-expression levels by qPCR may be of predictive value when assesing sensitivity to pemetrexed-based chemotherapy in NSCLC. There is an expanding corpus of reports about TYMS expression levels in NSCLC patients, some confirming that TYMS expression is significantly increased in tumor cells but not in normal epithelial cells [9, 13]. Recently, in accordance with the results found in our series described here, 2 meta-analyses reported that null or low expression of TYMS was associated with higher objective response in NSCLC patients treated with pemetrexed-containing chemotherapy [14, 15]. Of note, objective response rates were significantly higher in TYMS -/low expression patients than in TYMS +/high expression patients when examined by IHC. However, this difference did not reach statistical significance in studies performed by RT-PCR [14], thus contrasting with our results. Both of the aforementioned studies suggest that TYMS may be a suitable marker of sensitivity to pemetrexed-based chemotherapy in patients with NSCLC, although one of them indicates that the prognostic value of TYMS protein expression may need further validation. In our case, TYMS overexpression correlated significantly with progression and type of response. A small number of studies have also addressed the relationship between TYMS expression and effect of pemetrexed-based chemotherapy, although reports about the prognostic significance of TYMS expression in advanced NSCLC are controversial [16]. Notably, most of them suggest that elevated levels of TYMS expression are significantly associated with reduced tumor responses and shorter survival rates [6, 9, 17]. In addition, our data illustrated that treatment with pemetrexed benefited patients with low *TYMS* gene expression in terms of TTP. Although other studies have suggested that TYMS expression holds potential as a predictor of responsiveness to pemetrexed treatment in advanced cancer patients [3, 6, 9, 18], prospective studies are necessary to confirm these findings in NSCLC patients.

Our study offers evidence in support of using qPCR to determine TYMS mRNA expression as an alternative to the standard evaluation of protein expression (i.e., IHC), provided at least 80 % tumor cell content per sample is achieved by laser capture microdissection. Although most of the reported studies have used IHC to evaluate TYMS abundance levels, and only a minority have used qPCR, some meta-analyses have failed to find evidence of heterogeneity between detection-method subgroups (either IHC or qPCR) [14]. Moreover, another meta-analysis reported that there was a significant correlation between IHC and qPCR findings in the detection of TYMS expression and their corresponding associations with survival rates [3].

## Conclusions

In conclusion, our study carried out using qPCR assay reveals that the *TYMS* gene was predominantly overexpressed in these routine clinical samples, and that *TYMS* overexpression correlated with reduced response to pemetrexed-containing chemotherapy. In light of these findings, *TYMS* gene expression might be used as a predictive biomarker of sensitivity to pemetrexed-based chemotherapy in advanced NSCLC patients. Further prospective studies are ongoing in our institution to validate the appropriateness of using TYMS in clinical decision making.

## Abbreviations

TYMS: Thymidylate synthase
qPCR: Quantitative real-time PCR
FFPE: Formalin-fixed paraffin-embedded
IHC: Immunohistochemistry
TTP: Time to progression
OS: Overall survival
ROC: Receiver operating characteristic
REMARK: Reporting Recommendations for Tumor Marker Prognostic Studies
